# Case in Which a Quantity of Laudanum Was Removed from the Stomach by Means of a Syringe

**Published:** 1824-02

**Authors:** Henry Payne


Art. IV.—*Case in which a quantity of Laudanum was removed from the Stomach by means of a Syringe.* By Henry Payne, M.D.

The introduction into medical practice of an instrument, by which any quantity of laudanum received into the stomach may be removed from it, entitles those who have brought it into use to a share of the honours of the profession, perhaps equal to what has been bestowed on the most illustrious names that occur in the records of medicine.

I believe there are two gentlemen (Mr. Jukes, of London, and Mr. Bush, of Frome,) now living, who have an equal claim to the merit of bringing before the medical public the apparatus by which liquid laudanum may be effectually and expeditiously removed from the stomach.

A few months ago, Mr. Ward, chemist and druggist of this town, brought to my house for inspection what he called Jukes's apparatus, every part of which he explained the use of, in an intelligent and satisfactory manner. In consequence of this, and not knowing of the instrument being in the possession of any of the surgeons of Nottingham, I requested him, at about half-past eight o'clock of the evening of the 19th ultimo, to go down to New Snenton, a village a short distance from this town, to remove a quantity of laudanum from the stomach of a woman, whom I had just left in a state of stupor, from the effect of between an ounce and a half and two ounces, which I had ascertained she had taken, about seven hours previously to my seeing her, for the purposes of self-destruction. Mr. Ward, not wishing to apply the instrument alone, requested Mr. R. Williams, surgeon's pupil of this town, to accompany him, by whose assistance the elastic tube was introduced into the stomach, and through it nearly a quart of warm water was injected by means of the syringe, and then drawn up; the operation being continued until the liquid matter that was pumped up ceased to exhale the odour of opium.

It was not in my power to be present at the time the woman was undergoing the operation, but the contents of the stomach were brought to me, which were strongly impregnated with opium. Mr. Ward informed me, that the time occupied in clearing the stomach of the laudanum did not exceed ten minutes. At my visit on the following morning, (the 20th,) I found the woman in a state of languor and rather feverish, but quite recovered from the narcotic effects of the opium. The patient was thirty-five years of age, a married woman, the mother of several children, and far advanced in pregnancy.

After the operation, the patient took a little medicine of the febrifuge kind, to remove a degree of pyrexia, which was however very slight. She complained of no pain or difficulty in swallowing.

It is quite evident that the poison had remained upwards of seven hours in the stomach: nevertheless, so rapid was the recovery of the patient after its removal, that I found her below stairs on the morning of the 21st, following her usual avocations. It is also evident that the recovery of the patient must be entirely ascribed to the application of the apparatus, which, there is reason to believe, might be employed with equal success in cases wherein mineral, or any other poisons, have been received into the stomach.

In this instance of poisoning by laudanum, the patient being in the seventh month of her pregnancy, it is obvious that the application of the instrument was a far more appropriate remedy than powerful emetics, the repetition of which might have excited the action of the uterus, and produced abortion. The application of this new and truly valuable instrument took place, *in this instance*, two days prior to Sir Astley Cooper's experiment upon the dog, and is, I have reason to believe, the only case that has occurred in this part of the country, in which the apparatus has been employed.*

*Nottingham; Dec. 5th,* 1823.

* We beg to recommend the syringe of Mr. READ to the favourable notice of our readers: it appears to us extremly well adapted both for emptying the stomach and for throwing injections into the bowels.—EDITORS.

